# Supplementary material for: BGDMdocker: a Docker workflow for data mining and visualization of bacterial pan-genomes and biosynthetic gene clusters
Source: PeerJ. 2017 Nov 30;5:e3948. doi: 10.7717/peerj.3948 (PMC5712467; doi:10.7717/peerj.3948)
Supplement: Supplemental Information 1 [file peerj-05-3948-s001.docx]

**The command line and tip of BGDMdocker**

**1.Guide for BGDMdocker workflow usage：**

**1) [installing latest Docker-CE](https://docs.docker.com/engine/installation/linux/docker-ce/)** (Ubuntu, Debian, Raspbian, Fedora, Centos, Redhat, Suse, Oracle, Linux *et al*., all applicable):

$ curl -fsSL get.docker.com -o get-docker.sh

$ sudo sh get-docker.sh

adding user to the "docker" group with something like:

$ sudo usermod -aG docker <user name>

Type the following commands at your shell prompt. If this outputs the Docker version, your installation was successful:

$ docker version

On your host (with Docker), type the following command lines to build a BGDMdocker workflow:

$ git clone https://github.com/cgwyx/BGDMdocker.git

Or: download “[BGDMdocker-master.zip](https://github.com/cgwyx/BGDMdocker/archive/master.zip)” file

$ unzip BGDMdocker-master.zip

**2) Build workflow Images:**

$ cd ./BGDMdocker

$ docker build -t BGDMdocker:latest .

Or:pull Images of BGDMdocker from [DockerHub](https://hub.docker.com/r/cgwyx/bgdmdocker/),such as :

$ docker pull cgwyx/BGDMdocker

**3) Running Container and Prokka to genome annotation**

Copy the following commands to run the analysis for genome annotation of **Ba_xx** strains (for boldface text, please enter your data): **(if you have your own genome sequences, you need this step to generate** **“*.gbff” annotation files):**

$ docker run —rm -v home:home BGDMdocker \

prokka --kingdom Bacteria --gcode 11 --genus Bacillus \

--species **Amyloliquefaciens** \

--strain **Ba_xx** --locustag **Ba_xx** --prefix **Ba_xx** --rfam \

--rawproduct --outdir **/home/manager/PRJNA291327** \

**/home/manager/Ba_xx.fasta**

“**Ba_xx.fasta**” is the genome sequence and “**PRJNA291327**” is the output folder of results must be in your host.

**4) Running panX analysis pan-genome in Container** **of BGDMdocker in Command line** **interaction patterns.**

PanX starts with a set of annotated sequences files, *.gbff (.gbk) (e.g., NCBI RefSeq or GenBank),and these data should also reside in a folder within“ /pan-genome-analysis/data/ ”

in Container,we will refer to this folder as run directory below. The name of the run directory is used as a species name in down-stream analysis and visualization.Therefore,you need to enter the Container to run the relevant commands,and commit the Container to save image of visualization at last,Copy the following commands to run the analysis of the pan-genome of 44 *B. amyloliquefaciens* strains from the command-line interface of Container (for boldface text, please enter your data). For detailed parameters see [here](https://github.com/neherlab/pan-genome-analysis).

$ cd /pan-genome-analysis

$ cp -r /home/manager/B_amy /pan-genome-analysis/data/

$ ./panX.py -fn./data/B_amy -sl B_amy-RefSeq.txt -t 4

“/home/manager/B_amy” is your loclhost annotated sequences files of “*.gbff ”(GenBank files) and B_amy-RefSeq.txt (accession list for strains), should copy to Container and reside in “/pan-genome-analysis/data/B_amy” folder, The result will also be output to the this folder in Container.

Visualization of the pan-genome of 44 *B. amyloliquefaciens* strains (run in Container):

$ python link-to-server.py B_amy

$ add-new-pages-repo.sh B_amy

$ gulp

On you host ,open http://localhost:8000/B_amy with a web browser to access the visualization of the pan-genome immediately.

Create a new Image from Container for saving changes of visualization data (running in host):

$ docker commit <ID of Container > <name of new Image >

**5) Running Container and antiSMASH to search for gene clusters:**

Copy the following commands to run the analysis of biosynthetic gene clusters of Y2 strain from the command-line interface of Container (Y2.gbff) (for boldface text, please enter your data):

$ docker run —rm -v home:home BGDMdocker:latest \

run_antismash.py /home/manager/**Y2.gbff** \

--outputfolder /home/manager/**Y2_out** \

--input-type nucl --taxon bacteria \

--dbgclusterblast \

/antismash-4.0.0/antismash/generic_modules/clusterblast \

--pfamdir \

/antismash-4.0.0/antismash/generic_modules/fullhmmer \

--knownclusterblast --clusterblast --subclusterblast \

--borderpredict --transatpks_da \

--smcogs --inclusive --full-hmmer --asf

“*.gbff ”(GenBank files) is the genome sequence and “Y2_out” is the output folder of results must be in your host.

**2. Building workflow using standalone Dockerfile (recommendation):**

In order to meet the needs of different users, we also provide a standalone Dockerfile for Prokka, panX, and antiSMASH. You can build images and run Container separately.

**1) Building Image and run Container of Prokka standalone:**

$ git clone https://github.com/cgwyx/prokka_conda_docker.git

Or: download [“prokka_conda_docker-master.zip](https://github.com/cgwyx/prokka_conda_docker/archive/master.zip)” file

$ unzip prokka_conda_docker-master.zip

$ cd ./prokka_conda_docker-master

$ docker build -t conda:prokka .

Or: pull Image from [DockerHub](https://hub.docker.com/r/cgwyx/prokka_conda_docker/):

$ docker pull cgwyx/prokka_conda_docker

Run a Container from the image and copy the following commands to run the analysis of the genome annotation of **Ba_xx** strains (for boldface text, please replace with your own data if applicable):

$ docker run —rm -v home:home prokka:latest \

prokka --kingdom Bacteria --gcode 11 --genus Bacillus \

--species **Amyloliquefaciens** \

--strain **Ba_xx** --locustag **Ba_xx** --prefix **Ba_xx** --rfam \

--rawproduct --outdir /home/manager/**PRJNA291327** \

/home/manager/**Ba_xx.fasta**

“**Ba_xx.fasta**” is the sequence of the genome; “**PRJNA291327**” is the output folder of the results,thye are all must be in your host.

**2) Building Image and run Container of panX standalone:**

$ git clone https://github.com/cgwyx/panx_conda_docker.git

Or: [download](https://github.com/cgwyx/my_panx_conda_docker/archive/master.zip) “.zip” file

$ unzip panx_conda_docker-master.zip

$ cd ./panx_conda_docker-master

$ docker build -t conda:panx .

Or:pull Image from [dockerhub](https://hub.docker.com/r/cgwyx/panx_conda_docker/):

$ docker pull cgwyx/panx_conda_docker

$docker run -it --rm -v /home:/home cgwyx/panx_conda_docker:latest

Copy the following commands to run the analysis of the pan-genome of 44 *B. amyloliquefaciens* strains from the command-line interface of Container (for boldface text, please replace with your own data if applicable) ,you need to enter the container to run the relevant commands:

First your must copy data from your host to “/pan-genome-analysis/data/”,then run panX in Container;

$ cp -r /home/manager/**B_amy** /pan-genome-analysis/data/

$ ./panX.py -fn ./data/**B_amy** -sl **B_amy-RefSeq.txt** -t 4

*.gbff (GenBank files) and B_amy-RefSeq.txt (accession list for strains) should be in the “./data/B_amy” folder; output results will be also in “./data/B_amy” folder.

3. Visualize the pan-genome of 44 *B. amyloliquefaciens* strains (run in Container):

$ python link-to-server.py B_amy

$ add-new-pages-repo.sh B_amy

$ gulp

Open <http://localhost:8000/B_amy> with a web browser to access the visualization of the pan-genome immediately.

Create a new Image for saving changes in Container data of visualization(running in host):

$ docker commit <ID of Container > <name of new images >

**3) Building Image and run Container of antiSMASH4** **with** **database standalone:**

$ git clone https://github.com/cgwyx/antismash4_db.git

Or: [download](https://github.com/cgwyx/antismash4_lite_docker/archive/master.zip) “[antismash4_db-master.zip](https://github.com/cgwyx/antismash4_db/archive/master.zip)” file

$ unzip antismash4_db-master.zip

$ cd ./antismash4_lite_docker-master

$ docker build -t conda:antismash4_db .

Or: pull Image from [dockerhub](https://hub.docker.com/r/cgwyx/antismash4_db/):

$ docker pull cgwyx/antismash4_db

Run a Container from the image Copy the following commands to run the analysis of biosynthetic gene clusters of Y2 strain from the command-line interface of Container (Y2.gbff) (for boldface text, please replace with your own data if applicable):

$ docker run -it --rm -v home:home cgwyx/antismash4_db:latest \

run_antismash.py /home/manager/input/**Y2.gbff** \

--outputfolder /home/manager/output/**Y2_out** \

--input-type nucl --taxon bacteria \

--dbgclusterblast ./antismash/generic_modules/clusterblast \

--pfamdir ./antismash/generic_modules/fullhmmer \

--knownclusterblast --clusterblast --subclusterblast \

--borderpredict --transatpks_da \

--smcogs --inclusive --full-hmmer --asf

*.gbff (GenBank files) reside in “input”folder; “Y2 _out” is the output folder for the results(must be in your host).

**Tip: How can I download all “genomic.gbff.gz” of a specified species from the RefSeq or GenBank databases? Replace boldface text with your species if applicable:**

**1. Installing script on your host**

$ wget [ftp://ftp.ncbi.nlm.nih.gov/entrez/entrezdirect/versions/](ftp://ftp.ncbi.nlm.nih.gov/entrez/entrezdirect/versions/current/edirect.zip)

[current/edirect.zip](ftp://ftp.ncbi.nlm.nih.gov/entrez/entrezdirect/versions/current/edirect.zip)

$ unzip -u -q edirect.zip

$ export PATH=$PATH:$HOME/edirect

$ ./edirect/setup.sh

**2. Download “*genomic.gbff.gz” of all strains of *Bacillus amyloliquefaciens* from GenBank**

$ esearch -db assembly -query "Bacillus amyloliquefaciens [ORGN]" | efetch -format docsum | xtract -pattern "DocumentSummary" -element FtpPath_GenBank | sed 's/$/\/*genomic.gbff.gz/' |xargs wget -c -nd;sleep 3s;

**3. Download “*genomic.gbff.gz” of all *Bacillus amyloliquefaciens* strains from RefSeq**

$ esearch -db assembly -query "Bacillus amyloliquefaciens[ORGN]" | efetch -format docsum | xtract -pattern "DocumentSummary" -element FtpPath_RefSeq | sed 's/$/\/*genomic.gbff.gz/' |xargs wget -c -nd;sleep 3s;

**Visualizing results (local host)**

**For visualizing the pan-genome of 44 *B. amyloliquefaciens* strains on your loclhost like our [website](http://bapgd.hygenomics.com/pangenome/home) using Docker (Docker must be installed):**

Access the web download page at <http://bapgd.hygenomics.com/pangenome/home>and download the file “B_amly_44_strans_pan_genome_panx_vis.tar”,store in a home directory of your host. Copy the following commands to visualize the pan-genome of 44 *B. amyloliquefaciens* strains on the local host (Docker must be installed):

$ docker load < B_amly_44_strans_pan_genome_panx_vis.tar

$ docker run -d --rm -p 8000:8000 busybox_nodejs:nodejs_v7.3.0

Open http://localhost:8000/bamf_gbk44 with a web browser to access the visualization of pan-genome of 44 *B. amyloliquefaciens* strains immediately.

**For visualizing** **biosynthetic gene clusters** **of 44 *B. amyloliquefaciens* strains:**

Access the web download page at <http://bapgd.hygenomics.com/pangenome/home> and download data of genecluster of all strains,and you may download the standalone strain genecluster at home page also.Extract it into any directory then into the strain folder. Use the browser to open “index.html” to visualize the clusters of the strains.
